# Navigating the Impact of India’s Recent Ban on Fixed-Dose Combinations: A Call for Evidence-Based Regulation

**DOI:** 10.34172/apb.43741

**Published:** 2024-09-24

**Authors:** Muhammed Favas KT, Guru Datt Sharma, Sanjit Sah

**Affiliations:** ^1^Center for Global Health Research, Saveetha Medical College and Hospital, Saveetha Institute of Medical and Technical Sciences, Saveetha University, Chennai, Tamil Nadu, India.; ^2^Department of Evidence for Policy and Learning, Global Center for Evidence Synthesis, Chandigarh, India.; ^3^Department of General Medicine, Graphic Era Institute of Medical Sciences, Dehradun, Uttarakhand, India.; ^4^Department of Paediatrics, Dr. D. Y. Patil Medical College, Hospital and Research Centre, Dr. D. Y. Patil Vidyapeeth, Pune, Maharashtra, India.; ^5^Department of Public Health Dentistry, Dr. D.Y. Patil Dental College and Hospital, Dr. D.Y. Patil Vidyapeeth, Pune, Maharashtra, India.

## Dear Editor,

 We are writing to address the recent and significant decision by the Union Ministry of Health and Family Welfare (MoHFW) to ban 156 fixed-dose combinations (FDCs) in India,^1^ a move that has sparked widespread discussion within the medical and pharmaceutical communities. The ban, effective from August 2, 2024, includes a wide array of drugs used for common ailments such as colds, fevers, bacterial infections, and fungal infections. This decisive action, underpinned by expert recommendations and thorough evaluations, raises important questions about the future of drug regulation, patient safety, and the pharmaceutical industry in India.

 The decision to ban these FDCs is not without precedent. In 2016, a similar crackdown led to the prohibition of 344 FDCs, following the recommendations of the Professor Kokate Committee.^2^ The rationale behind both bans is rooted in concerns over the irrationality of these drug combinations and the associated risks to human health. The Drugs Technical Advisory Board (DTAB), a key player in the current ban, has categorically stated that many of these combinations lack therapeutic justification and pose a potential threat to patients, thereby making their prohibition necessary in the larger public interest.

 The primary justification for the ban lies in the identified risks associated with these FDCs. The DTAB and the expert committee appointed by the Government of India have highlighted the lack of evidence supporting the efficacy and safety of these combinations. Many of the banned FDCs are described as irrational because they combine multiple active pharmaceutical ingredients without sufficient clinical evidence to support their combined use. For instance, several combinations involving antibiotics, analgesics, and antihistamines were found to have no added therapeutic benefit over their individual components but were associated with increased risks, such as adverse drug reactions, drug resistance, and unnecessary exposure to multiple drugs.

 Furthermore, the use of certain FDCs could potentially contribute to the growing problem of antibiotic resistance, a global public health crisis. By combining antibiotics with other drugs, the risk of inappropriate use increases, which can accelerate the development of resistant strains of bacteria. This concern is particularly relevant in India, where antibiotic resistance is already a significant challenge.^3^

 The impact of this ban on the pharmaceutical industry cannot be understated. Major pharmaceutical companies such as Sun Pharmaceuticals, Cipla, Dr. Reddy’s Laboratories, Torrent Pharmaceuticals, and Alkem Laboratories are among those affected. The banned FDCs include widely used combinations like mefenamic acid with paracetamol and omeprazole with dicyclomine, which are staples in the Indian market. The immediate prohibition of these products will likely result in substantial financial losses for these companies, as well as disruptions in the supply chain.

 Moreover, this ban sends a strong message to the pharmaceutical industry about the need for rigorous testing and evidence-based practices in drug formulation and marketing. It underscores the importance of regulatory oversight in ensuring that only safe and effective drugs are available to the public. Pharmaceutical companies will need to adapt by investing more in research and development to create products that meet the stringent standards set by regulatory bodies.

 From a public health perspective, the ban is a proactive measure aimed at protecting patients from the potential harms associated with irrational drug combinations. The availability of safer alternatives further justifies the prohibition of these FDCs. However, there is also a need to ensure that the transition away from these banned drugs is managed carefully to avoid any disruption in patient care. Health professionals must be adequately informed about the ban and the alternatives available so that they can guide their patients appropriately.

 It is also crucial to consider the long-term effects of such bans on patient behavior and trust. Patients who have been using these FDCs for years may be resistant to change or may not fully understand the reasons behind the ban. Effective communication strategies are essential to ensure that patients are informed about the risks of these drugs and the benefits of switching to safer alternatives.

 The ban on 156 FDCs marks a significant step in the ongoing effort to regulate the pharmaceutical market in India and ensure patient safety. However, it also highlights the challenges of balancing public health concerns with the interests of the pharmaceutical industry. Going forward, there is a need for continued vigilance in the regulation of drug combinations and a commitment to evidence-based practices in drug development.

 It is also important for the regulatory authorities to work closely with the pharmaceutical industry to foster innovation while ensuring that patient safety remains the top priority. This could involve providing clearer guidelines for the development of FDCs and encouraging the use of combination therapies that have demonstrated clinical benefits.

 In conclusion, while the ban on 156 FDCs is a necessary and commendable action to protect public health, it also serves as a reminder of the complexities involved in drug regulation. It calls for a collaborative approach between regulators, the pharmaceutical industry, and healthcare professionals to ensure that the drugs available in the market are both safe and effective.

## Competing Interests

 None declared.

## Ethical Approval

 Not applicable.
